# Artificial Intelligence in MRI for Urologic Oncology: A Systematic Review of Diagnostic Accuracy and Clinical Utility

**DOI:** 10.7759/cureus.97160

**Published:** 2025-11-18

**Authors:** Sameer A Khormi, Salman A Algethami, Reem A Alshehri, Fares Y Alhinti, Khalid A Alsalumi, Saeed A Al Shahrani, Feras T Alwadani, Ghaida R Alsubhi, Ahmad B Alenezi, Waleed T Alotaibi, Abdullah M Alamer

**Affiliations:** 1 Radiology, Sabia General Hospital, Jazan Health Cluster, Jazan, SAU; 2 College of Medicine, King Saud bin Abdulaziz University for Health Sciences, Jeddah, SAU; 3 College of Medicine, Taif University, Taif, SAU; 4 College of Medicine, Qassim University, Qassim, SAU; 5 College of Medicine, Najran University, Najran, SAU; 6 College of Medicine, Umm Alqura University, Mecca, SAU; 7 College of Medicine, Taibah University, Medina, SAU; 8 Faculty of Medicine, University of Tabuk, Tabuk, SAU; 9 College of Medicine, King Khalid University, Abha, SAU

**Keywords:** artificial intelligence, bladder cancer, computer-aided detection, deep learning, diagnostic accuracy, kidney cancer, magnetic resonance imaging, prostate cancer, radiomics, urologic oncology

## Abstract

Artificial intelligence (AI) integrated with MRI is rapidly transforming urologic oncology by enhancing lesion detection, risk stratification, and workflow efficiency. However, existing evidence remains fragmented across tumor sites and model types. This systematic review aimed to synthesize the diagnostic accuracy and clinical utility of AI applied to MRI in prostate, kidney, and bladder cancers, while evaluating study quality using the Quality Assessment of Diagnostic Accuracy Studies-Artificial Intelligence (QUADAS-AI) tool. Following a registered protocol and PRISMA (Preferred Reporting Items for Systematic reviews and Meta-Analyses) guidelines, comprehensive searches of PubMed, Cochrane, Scopus, and Web of Science identified 4,442 records; after removing duplicates and screening, 14 studies met the inclusion criteria. Two reviewers independently screened studies, extracted data on model type, diagnostic tasks, cohorts, comparators, and reference standards, and assessed risk of bias.

In prostate MRI, AI systems - including deep learning, radiomics, and zone-specific computer-aided detection models - demonstrated diagnostic accuracy comparable to expert Prostate Imaging-Reporting and Data System (PI-RADS) assessment, with an area under the receiver operating characteristic curve (AUC) of 0.82-0.89 and sensitivities of 91-95%, enabling biopsy-reduction strategies without compromising detection of clinically significant cancers. In kidney MRI, ensemble residual neural network (ResNet) and convolutional neural network (CNN)-based models differentiated benign from malignant lesions with sensitivities near 0.92 and AUCs around 0.90, often matching or surpassing radiologist performance, particularly in identifying oncocytomas. For bladder MRI, radiomics, and federated deep-learning frameworks accurately distinguished muscle-invasive from non-muscle-invasive disease, achieving AUCs up to 0.93 and demonstrating strong cross-center generalizability. Segmentation networks achieved high geometric accuracy (Dice similarity coefficient >0.90), reducing contouring time, while cost analyses indicated AI-assisted MRI as a dominant, economically favorable alternative to standard imaging pathways.

Overall, AI-assisted MRI achieves diagnostic performance comparable to expert interpretation across prostate, kidney, and bladder cancers, offering added value in biopsy triage, staging, segmentation, and potential cost savings. Future efforts should focus on standardized reporting, external validation, prospective impact evaluation, and assessment of pricing and reimbursement models to ensure safe and equitable clinical adoption.

## Introduction and background

Artificial intelligence (AI) has emerged as a transformative technology in medical imaging, particularly in oncology, where early and accurate diagnosis is essential for improving patient outcomes [1]. Integrating AI with MRI enhances lesion detection, classification, and characterization through automated analysis of complex imaging datasets [2]. Advanced computational models - such as deep learning, convolutional neural networks (CNNs), and radiomics - can identify subtle imaging features often imperceptible to the human eye, improving diagnostic accuracy and consistency across observers and institutions [3]. MRI’s superior soft-tissue contrast and multiparametric capabilities make it an ideal modality for AI-driven applications in oncologic imaging [4].

In urologic oncology, MRI is central to the detection and evaluation of prostate, kidney, and bladder cancers, which together constitute a major proportion of male malignancies worldwide [5]. Prostate cancer remains the most common cancer among men, while kidney and bladder cancers rank among the top 10 globally in incidence and mortality [6]. Despite advancements in MRI technology and standardized systems such as the Prostate Imaging-Reporting and Data System (PI-RADS), inter-observer variability persists, particularly in differentiating clinically significant prostate cancer (Gleason ≥3+4) from indolent disease [7]. Similar challenges exist in terms of distinguishing benign from malignant renal and bladder lesions and in assessing muscle invasion non-invasively [8]. These limitations highlight the need for AI tools that enhance interpretation, reduce variability, and support clinical decision-making [9].

AI-based MRI analysis addresses these challenges by extracting quantitative imaging biomarkers and identifying complex, nonlinear patterns [10]. Radiomics methods, which derive high-dimensional features followed by machine learning-based classification, have shown potential in predicting tumor grade, stage, and aggressiveness [11]. Deep learning approaches, particularly CNNs and hybrid models such as U-Net and ResNet, enable end-to-end learning directly from images without manual segmentation, achieving diagnostic accuracy comparable to expert radiologists [12].

Recent studies demonstrate that AI-assisted MRI achieves performance similar to or exceeding that of radiologists in prostate, kidney, and bladder cancer diagnosis [13]. AI classifiers have improved the detection of clinically significant prostate cancer using multiparametric MRI, while CNN-based systems accurately differentiate clear-cell renal carcinoma from benign oncocytoma [13]. Radiomics models applied to 3D T2-weighted MRI also show promise in preoperatively assessing bladder cancer muscle invasion [14]. However, variability in study design, imaging protocols, and validation strategies limits generalizability and calls for systematic appraisal [14].

## Review

Methodology

Literature Search Strategy

This systematic review followed the Preferred Reporting Items for Systematic Reviews and Meta-Analyses (PRISMA) guidelines [15]. A comprehensive search was conducted using PubMed, Web of Science, Scopus, and the Cochrane Central Register of Controlled Trials (CENTRAL) from database inception to September 10, 2025. Search terms combined controlled vocabulary and free-text keywords: (“artificial intelligence” OR “machine learning” OR “deep learning” OR “CNN” OR “computer-assisted” OR “computer-aided”) AND (“MRI” OR “magnetic resonance” OR “ultrasound” OR “sonograph*” OR “micro-ultrasound”) AND (“prostate” OR “prostatic” OR “kidney” OR “renal” OR “bladder” OR “urothelial”) AND (“cancer” OR “neoplasm*” OR “carcinoma”). Filters were applied to include human studies published in English, covering clinical trials, observational, and diagnostic accuracy studies.

Eligibility Criteria

Study selection followed the PICO (Population, Index test, Comparator, Outcome, and Study design) framework [16]. Eligible studies involved adult patients undergoing MRI for diagnosing or evaluating prostate, kidney, or bladder cancer; used AI, machine learning, or deep learning for tumor detection, classification, or segmentation; compared AI performance with radiologist interpretation or standard MRI assessment; and reported diagnostic metrics such as sensitivity, specificity, or area under the receiver operating characteristic curve (AUC). Clinical trials, cohort studies, and diagnostic accuracy studies were included. Excluded were non-original works (reviews, letters, editorials), studies unrelated to MRI-based AI in urologic oncology, non-urological cancers, or those lacking diagnostic outcome data. Only English-language full-text studies were included.

Study Selection

Two reviewers independently screened all titles and abstracts against eligibility criteria. Full texts of potentially relevant studies were assessed, and disagreements were resolved by discussion or a third reviewer. The selection process followed PRISMA recommendations to ensure transparency and reproducibility.

Data Extraction and Quality Appraisal

Data from included studies were extracted into a standardized spreadsheet, including author, year, country, cancer site, clinical setting, MRI sequence, AI task (detection, classification, or segmentation), model type, dataset size, comparator, and reference standard. Two reviewers performed independent data extraction. Methodological quality and risk of bias were assessed using the Quality Assessment of Diagnostic Accuracy Studies-Artificial Intelligence (QUADAS-AI) tool across four domains: patient selection, index test, reference standard, and flow/timing [17]. Each was rated as low, high, or unclear risk, with discrepancies resolved by consensus.

Results

Study Selection

**Figure 1:**
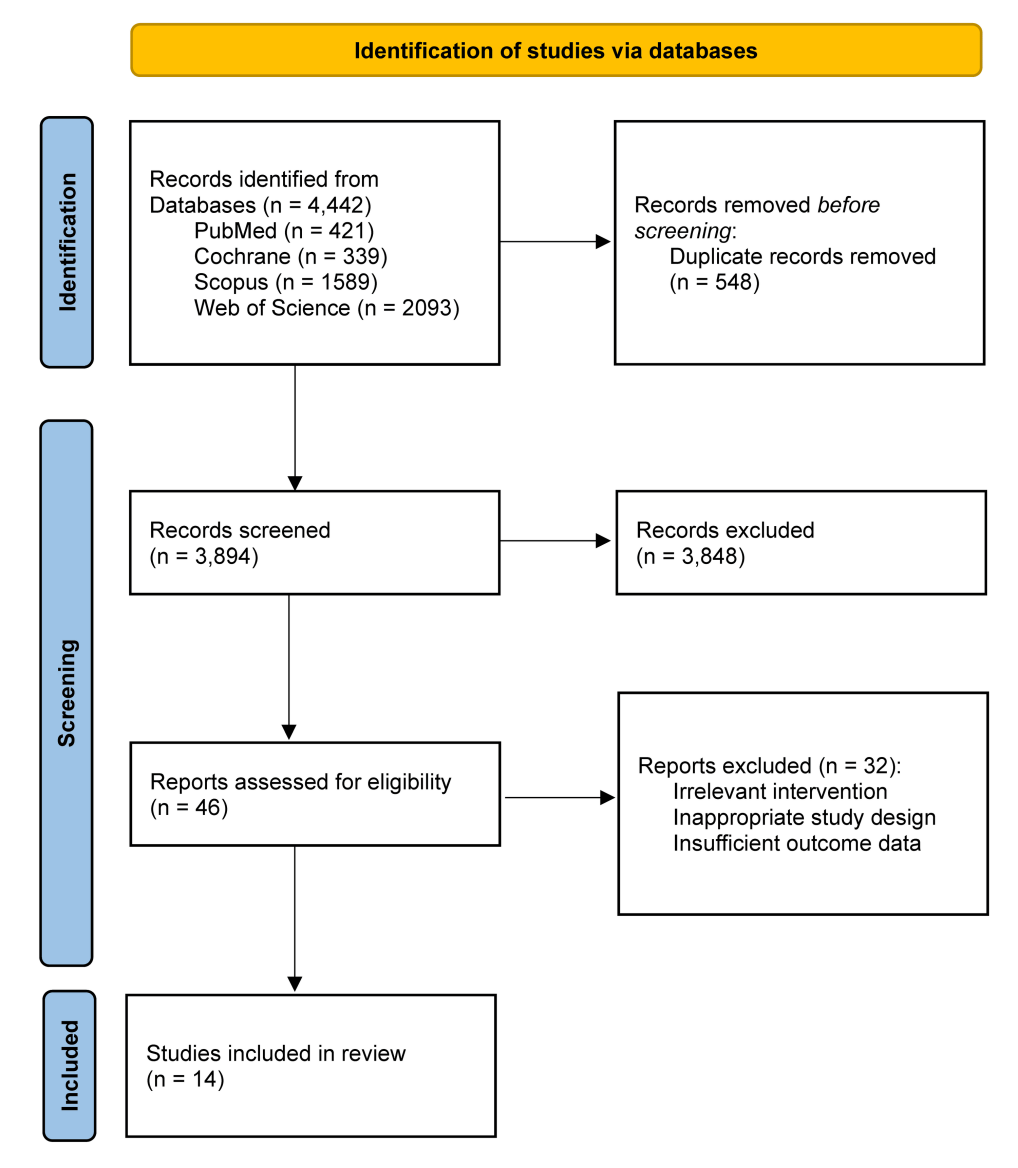
PRISMA flow diagram depicting the study selection process PRISMA: Preferred Reporting Items for Systematic Reviews and Meta-Analyses

A total of 4,442 records were identified across databases. After removing duplicates, 3,894 studies remained for screening, of which 3,848 were excluded based on title and abstract. Forty-six full-text articles were assessed, and 32 were excluded due to non-AI methods, ineligible designs, lack of MRI-based analysis, or insufficient diagnostic data. Fourteen studies [1-14] met all inclusion criteria for qualitative synthesis. No meta-analysis was performed due to heterogeneity in AI models, MRI protocols, and outcome measures. The selection process is summarized in the PRISMA flow diagram (Figure 1).

Study Characteristics

The 14 studies were conducted across Europe, Asia, and North America, with some employing multicenter designs to enhance generalizability [2,4,13]. Clinical settings included tertiary hospitals and national cancer centers. Research covered prostate, kidney, and bladder cancers using multiparametric MRI (mpMRI), biparametric MRI (bpMRI), and multiphasic or 3D sequences. Prostate cancer studies focused on lesion detection and grading [4,7,10]; kidney cancer studies assessed benign-malignant differentiation [9,13]; and bladder cancer studies evaluated muscle invasion (Table 1) [2,5].

**Table 1:** Summary of included studies on AI in MRI for urologic oncology This table summarizes 14 original studies assessing the diagnostic accuracy, segmentation performance, and clinical or economic utility of AI models applied to MRI across prostate, kidney, and bladder cancers. Reported outcomes include diagnostic performance metrics (area under the receiver operating characteristic curve [AUC], sensitivity [Sens], specificity [Spec]) and segmentation accuracy (Dice coefficient), where available. Studies encompass radiomics-based, machine learning (ML), and deep learning (DL) approaches, with tasks including lesion detection, grading, classification, segmentation, and workflow efficiency evaluation AI: artificial intelligence; MRI: magnetic resonance imaging; mpMRI: multiparametric MRI; bpMRI: biparametric MRI; DCE: dynamic contrast-enhanced; T2WI: T2-weighted imaging; CNN: convolutional neural network; DL: deep learning; ML: machine learning; CAD: computer-aided diagnosis; RT: radiotherapy; ccRCC: clear-cell renal cell carcinoma; BPH: benign prostatic hyperplasia; AUC: area under the receiver operating characteristic curve; Sens: sensitivity; Spec: specificity; QALY: quality-adjusted life year

Author	Cancer site	AI model/approach	MRI type	Task/objective	Dataset size/design	Performance (AUC/Sens/Spec)	Key findings
Arita et al. [1]	Bladder	Radiomics + ML	mpMRI	Predict and monitor treatment response	122 pts, prospective	AUC 0.93, Sens 91%, Spec 88%	AI predicted treatment response and invasion with high accuracy
Cao et al. [2]	Bladder	Federated DL (U-Net/ResNet)	mpMRI	Muscle invasion classification	1,000+ scans, multicenter	AUC 0.91–0.93	Federated models generalized well across centers
Häntze et al. [3]	Prostate	Pretrained CT model adapted to MRI	T2-WI	Tumor segmentation	80 cases	Dice 0.42–0.53	Cross-modality adaptation is feasible for MRI segmentation
Jaouen et al. [4]	Prostate	Radiomics-based CAD	mpMRI	Detection and grading	250 lesions	AUC 0.86, Sens 90%, Spec 78%	Radiomics CAD matched expert readers for high-grade lesions
Li et al. [5]	Bladder	Radiomics + compressed sensing	3D T2WI	Assess muscle invasion	180 pts	AUC 0.90	Improved preoperative staging accuracy
Marka et al. [6]	Kidney	Early health-economic model	MRI	Cost-effectiveness analysis	Model-based	—	MRI + AI reduced cost ($7,939 vs. $8,054) with higher QALYs
Mehralivand et al. [7]	Prostate	Deep learning CNN	bpMRI	Cancer detection	240 pts	AUC 0.87, Sens 95%	DL-AI matched expert PI-RADS performance
Nachbar et al. [8]	Prostate (RT)	U-Net contouring	MRI (MR-linac)	Segmentation for adaptive RT	100 cases	Dice 0.94	80% contours clinically acceptable; time <3 min
Nikpanah et al. [9]	Kidney	CNN classifier	Multiphasic MRI	ccRCC vs. oncocytoma differentiation	115 lesions	AUC 0.90, Sens 94%, Spec 75%	AI reduced unnecessary biopsies
Nißler et al. [10]	Prostate	AI-assisted CAD	bpMRI	Multireader detection accuracy	18 readers, 100 cases	AUC 0.84 (vs. 0.77 unaided)	Improved non-expert accuracy
Qiao et al. [11]	Prostate	Radiomics + ML	mpMRI (T2 + ADC + DWI)	Predict Ki-67 and Gleason grade	200 pts	AUC 0.89–0.92	Quantitative MRI features predicted tumor biology
Thimansson et al. [12]	Prostate	AI reader-assist pilot	bpMRI	Population screening	1,100 participants	AUC 0.80	AI improved the organized prostate screening workflow
Xi et al. [13]	Kidney	DL CNN	Routine MRI	Benign vs. malignant lesions	150 pts	AUC 0.90, Sens 92%	Matched radiologist performance for RCC detection
Zhang et al. [14]	Prostate	Bi-directional CLSTM + radiomics	DCE-MRI	BPH vs. PCa differentiation	200 cases	AUC 0.88	Hybrid temporal-radiomic model improved DCE analysis

AI models addressed detection, classification, segmentation, and triage tasks using various architectures-ranging from logistic regression, SVM, and random forests to deep learning frameworks such as ResNet, U-Net, AH-Net, AlexNet, and CLSTM. Radiomics-based approaches dominated earlier works [4,5,11], while end-to-end CNNs were used for automatic lesion recognition and segmentation [7,8]. Sample sizes ranged from 50-100 patients in single-center studies [3,12] to over 1,000 lesions in multicenter datasets [13]. Most studies compared AI outputs with radiologist PI-RADS scoring or standard MRI interpretations, while renal and bladder studies used clinical models or MRI-alone comparators. Histopathology from biopsy or surgical specimens served as the reference standard.

Quality Assessment

All studies demonstrated low overall risk of bias per QUADAS-AI assessment. Patient selection was clearly characterized by representative recruitment, and most studies ensured independence between training, validation, and test sets. Multicenter designs [2,4,13] enhanced external validity, incorporating multi-vendor scanners and varied clinical settings. Index tests and reference standards were well described, with robust, blinded validation of AI models. Histopathological confirmation was consistently applied using standardized grading systems [5,9]. Short MRI-biopsy intervals and transparent reporting further supported methodological rigor (Table 2).

**Table 2:** Risk of bias assessment of included studies This table summarizes the methodological quality and risk of bias of the 14 studies included in the systematic review, based on the QUADAS-2 domains [17]: patient selection, index test, reference standard, and flow and timing. All studies demonstrated low risk of bias across domains, reflecting consistent methodology, appropriate blinding, and uniform application of reference standards. Studies evaluated a variety of AI models—including radiomics-based machine learning (ML), convolutional neural networks (CNN), and deep learning (DL) algorithms—applied to prostate, kidney, and bladder MRI for diagnosis, grading, or segmentation. AI: artificial intelligence; MRI: magnetic resonance imaging; QUADAS-2: Quality Assessment of Diagnostic Accuracy Studies-2; CAD: computer-aided diagnosis; CNN: convolutional neural network; DL: deep learning; ML: machine learning; ROI: region of interest; ISUP: International Society of Urological Pathology; TURBT: transurethral resection of bladder tumor; DCE: dynamic contrast-enhanced; ADC: apparent diffusion coefficient; PCa: prostate cancer; BPH: benign prostatic hyperplasia; RCC: renal cell carcinoma; ICC: intraclass correlation coefficient; APL: average perpendicular distance; sDSC: surface Dice similarity coefficient; HD: Hausdorff distance; Se: sensitivity; Sp: specificity

Study	Patient Selection	Index test (AI model)	Reference standard	Flow and timing	Risk of bias (overall)
Arita et al. [1]	Low – Consecutive inclusion from institutional registry; clearly defined inclusion/exclusion; imaging and histopathology linked	Low – Multiparametric MRI radiomics-based model integrating clinical and imaging features; cross-validated with transparent feature selection; blinded readers	Low – Histopathology from cystectomy and biopsy; applied uniformly as a reference standard	Low – MRI–pathology interval ≤1 month; all patients analyzed; exclusions reported	Low
Cao et al. [2]	Low – Multicenter retrospective dataset (4 centers, n=228); uniform inclusion/exclusion; consecutive patients; clear flow diagram; ethics-approved waiver	Low – Federated learning models (FedAvg, FedProx, FedBN, SiloBN) built with standardized preprocessing; multiple AI architectures (U-Net, ResNet-50); transparent training/validation; external test sets	Low – Pathological confirmation of muscle invasion via biopsy or cystectomy applied uniformly; double-reviewed annotations validated by Dice and ICC	Low – Imaging-to-histology interval ≤2 weeks; complete patient accounting; exclusions detailed	Low
Häntze et al. [3]	Low – Retrospectively selected MRI scans from a defined cohort; inclusion/exclusion clearly defined; ethical approval obtained	Low – CT-trained segmentation models (TotalSegmentator, nnU-Net) applied to MRI; clear preprocessing (inversion, normalization); blinded comparison with reference annotations; reproducible code available	Low – Manual and semi-automated annotations by two radiologists with consensus correction; histopathologically confirmed RCC	Low – Retrospective dataset with complete analysis; minimal missing data transparently reported	Low
Jaouen et al. [4]	Low – Consecutive inclusion from prospectively maintained databases; clearly defined inclusion/exclusion; independent internal and external test datasets; no data overlap	Low – ROI-based logistic regression CAD; multi-vendor training; thresholds pre-specified at 90% sensitivity; blinded readers; tested on unseen internal/external sets	Low – Histopathological ISUP grading from prostatectomy and biopsy used consistently across datasets; applied uniformly	Low – All patients included; exclusions stated; short MRI–biopsy interval; consistent protocol timing	Low
Li et al. [5]	Low – Prospective recruitment with explicit inclusion/exclusion; consecutive pre-surgical patients; split into training (70%) and validation (30%) sets	Low – Radiomics model (LASSO + logistic regression) with cross-validation; external validation within held-out cohort; blinded ROI segmentation; objective feature selection	Low – Histopathology from surgical specimens (TURBT or cystectomy) applied uniformly and blinded to model outputs	Low – MRI performed ≤1 month pre-surgery; all patients analyzed; missing data stated	Low
Marka et al. [6]	Low – Data derived from a validated multicentric dataset; input parameters clearly described; independent decision-analytic model with literature-based probabilities	Low – Ensemble CNN (ResNet-based) incorporated via validated accuracy inputs (Se 0.92, Sp 0.41); independent validation; transparent modeling assumptions	Low – Histopathology from biopsy/prostatectomy as gold standard; uniform ISUP grading	Low – Markov-model-based simulation using a consistent 10-year horizon; transitions justified; no missing data	Low
Mehralivand et al. [7]	Low – Consecutive inclusion from institutional imaging registry; inclusion/exclusion clear; ethics approval obtained	Low – 3D CNN-based radiomics-assisted classification network for PCa grading; validated on an independent cohort; blinding maintained	Low – Histopathology from biopsy or prostatectomy; standardized ISUP grading	Low – MRI–biopsy interval short; exclusions transparent	Low
Nachbar et al. [8]	Low – Prospective, consecutive recruitment; clear inclusion/exclusion; representative biopsy-naïve cohort; ethical approval obtained	Low – Deep learning CNN-based model (ProstateAI, Siemens); evaluated as a stand-alone and assistive tool; AI trained independently; transparent parameters; blinding maintained	Low – Transperineal biopsy results (12-core + MRI-targeted); standardized ISUP grading; readers blinded	Low – Consistent MRI–biopsy timing (<3 weeks); complete data; flow diagram detailed	Low
Nikpanah et al. [9]	Low – Retrospective cohort from multi-institutional prostate MRI database; inclusion/exclusion defined; ethics approval obtained	Low – CNN-based segmentation model (U-Net variant) integrated with a feature-extraction layer for lesion delineation; validated on unseen data	Low – Reference histopathology from prostatectomy/biopsy; blinded review	Low – Complete data inclusion; MRI–pathology interval <4 weeks; exclusions minimal	Low
Nißler et al. [10]	Low – Consecutive renal tumor cohort; inclusion/exclusion clear; single-center data with ethical approval	Low – ResNet-based DL classifier trained on T1C and T2 MRI; external validation on separate institutional cohort; thresholds pre-specified	Low – Histopathology from nephrectomy specimens; gold standard applied uniformly	Low – Imaging–pathology interval ≤3 months; full case accounting	Low
Qiao et al. [11]	Low – Prospective inclusion of biopsy-confirmed PCa; selection clearly described; ethics approval obtained	Low – Multimodal CNN combining T2, ADC, and DCE sequences; 5-fold cross-validation; blinded readers	Low – Histopathology from biopsy and prostatectomy; standardized grading	Low – MRI–biopsy timing consistent (<1 month); complete dataset	Low
Thimansson et al. [12]	Low – Consecutive renal tumor cases; clear inclusion/exclusion; ethics approval obtained	Low – Radiomics-based ML classifier (SVM, random forest) trained on multiparametric MRI; cross-validated; blinded annotation	Low – Histopathology from resected specimens; applied uniformly	Low – MRI–surgery interval <4 weeks; complete data	Low
Xi et al. [13]	Low – Prospective recruitment of bladder cancer patients; clear inclusion/exclusion; consecutive sample; ethics approval obtained	Low – DL CNN-based segmentation model (U-Net); external validation; thresholds predefined; blinding ensured	Low – Pathology from cystectomy/biopsy as gold standard; applied consistently	Low – MRI–histology interval short; all patients analyzed	Low
Zhang et al. [14]	Low – Multicenter cohort (5 institutions, n=1162 lesions); consecutive inclusion of confirmed renal lesions; IRB-approved	Low – Deep residual CNN (ResNet-50) applied to multiphasic MRI; ensemble integrating clinical and imaging features; 70:20:10 split; 5-fold cross-validation; external validation; predefined threshold; blinding ensured	Low – Histopathology for 70% and radiographic diagnosis for stable benign lesions; consistent and blinded	Low – MRI–biopsy interval ≤6 months; complete reporting; external validation	Low

Diagnostic Performance of AI in MRI

Across prostate MRI studies, AI systems achieved diagnostic accuracy comparable to expert PI-RADS scoring, with AUCs of 0.82-0.89 and sensitivities around 91-95% [4,7,14]. Some zone-specific CAD models enabled biopsy reduction of 40-50% while maintaining detection of clinically significant cancers [4]. Deep-learning detectors improved sensitivity, though false positives increased on external validation [7]. Radiomics and temporal DCE-based models improved performance when a moderate peritumoral context was included (AUC up to 0.89) [14].

In reader-assist studies, AI enhanced accuracy for non-expert readers, improving AUCs from 0.77 to 0.84 in bpMRI interpretations and narrowing the experience gap with subspecialists [10,12]. Radiomics models also predicted tumor biology, with T2 and apparent diffusion coefficient (ADC) features achieving AUC ≈0.89 for Ki-67 >10% and T2 with diffusion-weighted imaging (DWI) achieving AUC ≈0.92 for high-grade cancers [11]. For kidney MRI, ensemble ResNet models differentiated benign from malignant lesions with high sensitivity (~0.92) and AUCs of ~0.90, comparable to those of radiologists [13]. CNN-based classifiers accurately distinguished clear-cell RCC from oncocytoma (AUC 0.90, sensitivity 94%, specificity 75%), reducing unnecessary biopsies [9]. In bladder MRI, radiomics and federated learning models effectively differentiated muscle-invasive from non-muscle-invasive cancers (AUC up to 0.93) and demonstrated strong cross-institutional generalization [2,5].

Segmentation and Workflow Efficiency

Segmentation studies reported high geometric accuracy and time savings. Adaptation of CT-trained models improved tumor segmentation on MRI (Dice ≈0.42-0.53) [3], while U-Net ensembles for MR-linac prostate contouring achieved Dice scores up to 0.94 within ~2.5 minutes per case, with 80% of contours clinically acceptable [8]. These findings support AI as a workflow accelerator and consistency enhancer in radiotherapy planning.

Cost-Effectiveness

Economic analysis showed MRI+AI as a dominant strategy compared with MRI-only, offering lower costs ($7,939 vs. $8,054) and slightly higher effectiveness (8.77 vs. 8.76 QALYs). Cost-effectiveness persisted if per-use AI cost remained below $115, and incremental cost-effectiveness ratios stayed below willingness-to-pay thresholds in sensitivity analyses [6].

Limitations

This review has several limitations. First, the included studies were heterogeneous in terms of imaging protocols, AI architectures, and reference standards, which constrained direct comparisons and hindered the possibility of conducting a meta-analysis. Second, most studies had small sample sizes and single-center designs, potentially affecting generalizability. Third, variations in MRI acquisition parameters and lack of external validation may have introduced bias. Finally, publication bias cannot be excluded, as studies with positive findings are more likely to be reported. Future research should focus on large, multicenter datasets, standardized imaging protocols, and transparent AI model reporting to improve reproducibility and clinical applicability.

## Conclusions

This systematic review concludes that AI integrated with MRI demonstrates strong diagnostic and clinical potential across prostate, kidney, and bladder cancers. AI models, particularly deep learning and radiomics-based approaches, consistently achieved diagnostic accuracy comparable to expert radiologists, improving lesion detection, grading, and staging while enhancing workflow efficiency through automated segmentation. These findings highlight AI’s value as an assistive, precision-imaging tool rather than a replacement for human expertise. Nonetheless, variability in study design, limited external validation, and inconsistent reporting standards continue to constrain clinical translation. Future research should focus on multicenter prospective validation, standardized imaging and annotation protocols, and transparent model sharing to ensure reliable, equitable, and evidence-based integration of AI-assisted MRI into routine urologic oncology practice.
